# Economic influences on population health in the United States: Toward policymaking driven by data and evidence

**DOI:** 10.1371/journal.pmed.1003319

**Published:** 2020-09-02

**Authors:** Atheendar S. Venkataramani, Rourke O’Brien, Gregory L. Whitehorn, Alexander C. Tsai

**Affiliations:** 1 Department of Medical Ethics and Health Policy, Perelman School of Medicine, University of Pennsylvania, Philadelphia, Pennsylvania, United States of America; 2 Leonard Davis Institute of Health Economics, University of Pennsylvania, Philadelphia, Pennsylvania, United States of America; 3 Department of Sociology, Yale University, New Haven, Connecticut, United States of America; 4 Center for Global Health and Mongan Institute, Massachusetts General Hospital, Boston, Massachusetts, United States of America; 5 Harvard Medical School, Boston, Massachusetts, United States of America; 6 Mbarara University of Science and Technology, Mbarara, Uganda

## Abstract

Atheendar S. Venkataramani and colleagues discuss economic factors and population health in the United States.

**Summary points**

- The United States is in the midst of a 40-year-long population health crisis. Life expectancy has declined since 2014, an unprecedented event that has followed on the heels of a decades-long slowing in secular gains in longevity in the US relative to peer countries. These adverse population health trends appear to be primarily driven by worsening health among working-age individuals of lower socioeconomic status.
- A growing body of research suggests that worsening economic outcomes—e.g., fading employment opportunities and increasing economic insecurity—may be a primary causal driver of adverse health trends among low-income and less-educated working-age US residents.
- Evidence-based public policies to address widening gaps in economic and health outcomes include expanding early childhood health and educational investments, increasing the scope of programs that assist displaced workers in developing new skills and finding new jobs, reinforcing the social safety net, and improving the reach of public health efforts to help moderate the health consequences of adverse economic shocks.
- Policymakers will also need to consider and rigorously evaluate new approaches, such as basic income grants, investments to direct automation toward complementing rather than replacing the work force, or job guarantee programs.
- The size and scope of the population health challenges that have arisen with the changing economy highlight the importance of new data sources and evidence-based engagement by policymakers.

## Introduction

Over the last 40 years, the secular increase in longevity in the US has slowed relative to peer countries, a phenomenon capped in more recent years by an unprecedented decline in US life expectancy [1]. These broad trends mask widening disparities in health outcomes by socioeconomic status. Stagnant—and more recently, rising—mortality among working-age adults in lower income brackets who have less formal education, primarily driven by rising drug overdose and suicide death rates, appear to entirely account for the growing gap in health outcomes between the US and other high-income countries [1–4].

During this time, economic outcomes in the same population subgroups have worsened as well. Since the late 1970s, growth in wages and incomes stagnated for most US residents [5]. The ability of individuals from poor families to achieve upward socioeconomic mobility has fallen considerably [6–8], whereas insecurity in many aspects of life—such as earnings, work, and housing—has risen [8,9]. Consequently, income inequality has increased dramatically over this period, reaching levels not seen since the eve of the 1929–1939 Great Depression [10].

We argue that worsening economic outcomes among low-income and less-educated working-age adults may be a key driver of adverse population health trends in the US. We begin by describing trends in economic outcomes since the late 1970s and their underlying drivers. We then discuss the myriad ways in which these trends have affected population health, focusing primarily on working-age adults. We review existing and new interventions that may jointly improve economic outcomes and population health (or mitigate the adverse health consequences of negative economic shocks). We also highlight the importance of new and more accessible data to better inform policy and the role of political processes in realizing the full potential of evidence-based policymaking.

## Worsening economic outcomes in the US

One of the most striking changes in the US economy has been the divergence, since 1980, in economic outcomes by level of education and relative position in the income distribution. Through the late 1970s, inflation-adjusted (i.e., real) wages grew at similar rates for US residents across the income distribution. Thereafter, however, real incomes stagnated for individuals in the bottom 50%, even as they continued to increase for those in the top 10% [5,11]. This divergence is even more stark when stratified by level of education. Over the same 40-year period, real earnings increased for those with a college education but decreased for those with only a high school education or less. At the same time, individuals growing up in poor families have found it increasingly difficult to exit poverty later in life. Economic opportunity—the ability to achieve upward socioeconomic mobility regardless of one’s background—has declined dramatically, particularly for those entering the US labor market in the early 1980s and thereafter [6,7].

Several forces may have contributed to these trends. The disappearance of employment opportunities that had previously provided to individuals without a college education a credible path to the middle class—e.g., manufacturing jobs—has played an outsized role [3,12,13]. These jobs have disappeared, in part, because of technological advances that have allowed firms to automate many tasks previously performed by workers [14] and also because of increases in foreign trade that have led to large declines in employment within industries and areas most exposed to competition with foreign firms [13,15]. The growth of low-wage healthcare industry jobs has buffered some areas against the loss of manufacturing employment opportunities but, overall, has done little to counter the worsening economic outcomes among low-income and less-educated workers that have been caused by automation and trade [16,17].

In addition to these market forces, shifts in public policy have also contributed to rising income inequality and falling social mobility in the US. Starting in the late 1970s, federal minimum wage increases have failed to keep up with inflation, and the inflation-adjusted minimum wage today is lower than it was in 1980. Recent studies have demonstrated the critical importance of minimum wage increases in sustaining incomes of low-wage workers, with attendant consequences for the shape of the overall income distribution [18,19]. Second, the deregulation of the financial industry has contributed to increasing “financialization” of the economy, which has both increased the relative importance of financial firms—and the wages of those employed within them—and exposed small businesses and individuals to greater financial risk [20]. The consequences of financialization are illustrated by the substantial declines in wealth among low- and middle-income households after the post-2007 Great Recession, compared with increasing wealth among higher-income households [21]. Third, emerging evidence suggests that deunionization has reduced the bargaining power of workers without college degrees, thereby undermining union-associated earnings premia and worsening income inequality [22,23].

Fourth, further entrenchment of structural racism has contributed to economic inequality by eroding economic outcomes among historically minoritized populations [24]. For example, after a period of narrowing, the gap in earnings between Black and White Americans has grown since the late 1970s [25]. Shifts in policing, criminal justice, and sentencing policies resulting in mass incarceration have contributed to this rise in economic inequality [26,27]. In addition, the persistence of racial segregation [28–30]—and the inability of local, state, and federal governments to mount definitive policy responses to effectively eliminate it [31]—has undermined the resilience of Black Americans to negative economic shocks [32], while remaining a continuing driver of poverty among Black Americans and Black-White disparities in income and wealth [33].

Policy-driven erosion of the social safety net has compounded the adverse socioeconomic consequences of the changing economy on low-income and less-educated individuals [34]. For example, cuts to cash welfare programs under both Republican and Democratic presidential administrations have reduced the share of poor families receiving benefits during times of need, from 4 in 5 families accessing welfare in 1980 to 1 in 5 in the present day [35]. The combination of falling wages, fading economic opportunities, and a shrinking safety net has introduced increasing precarity in the lives of the poor, a state marked by income insecurity and unpredictability, disengagement from social and economic structures, decreasing resilience to compounding social and economic shocks, and the need to make hard choices around basic needs [34,36–39].

## Economic outcomes and population health

### Theoretical mechanisms linking economic conditions and population health

**Fig 1:**
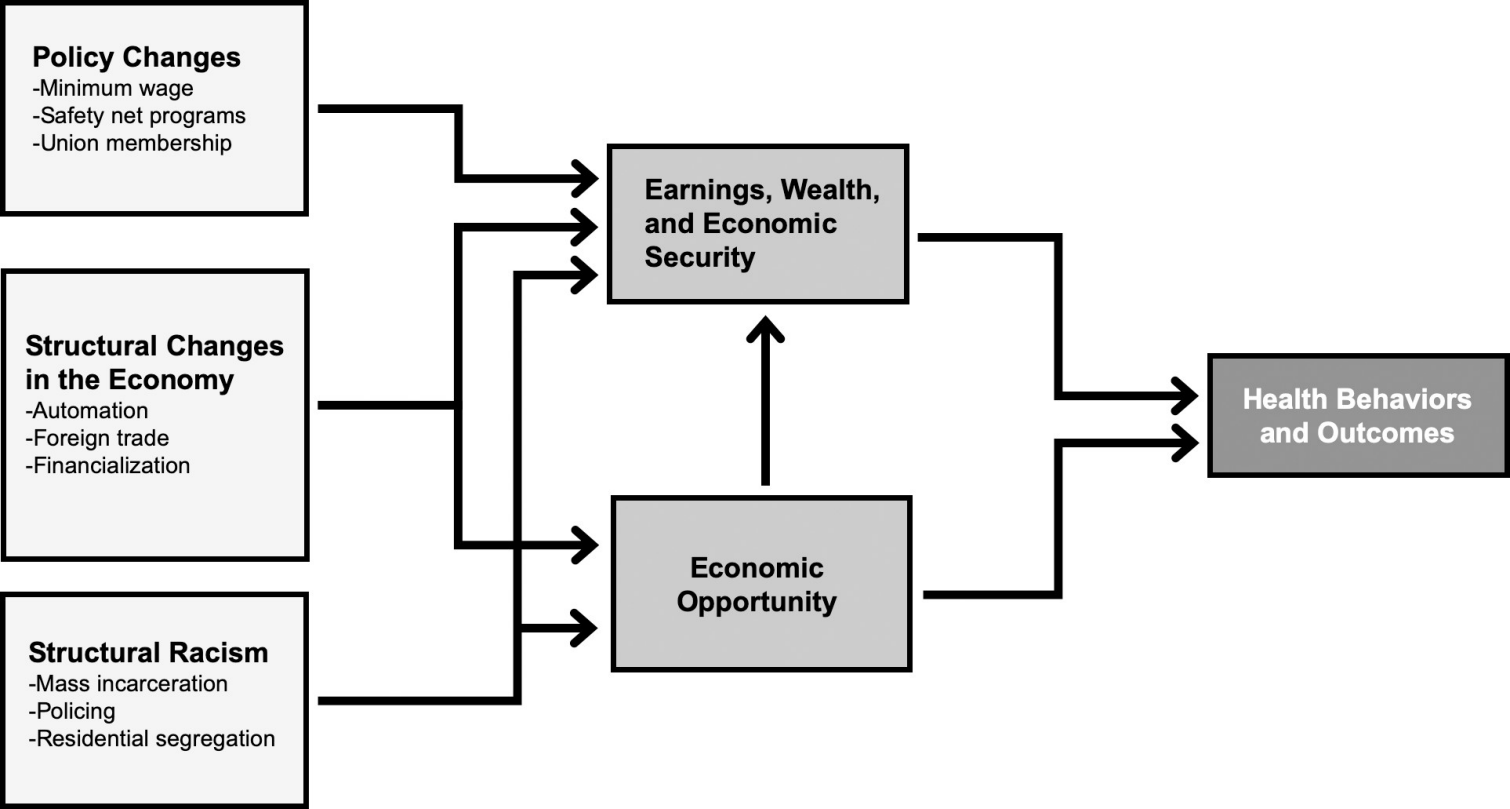
Drivers of economic outcomes and consequences for population health. Figure summarizes the key relationships between economic outcomes—and their underlying drivers—and health. To ensure better clarity, the figure only focuses on drivers and pathways discussed in this review. In doing so, we do not directly specify some relationships that are likely important for population health, e.g., direct effects of public policy (e.g., Medicaid expansions) and structural racism on health outcomes.

Worsening economic outcomes for less-educated and low-income individuals can influence health through several channels [2,40,41] (Fig 1). Falling incomes can reduce access to basic material resources (e.g., stable housing, food, health insurance, and healthcare) needed to ensure good health [41,42]. Worsening economic outcomes may also increase exposure to stressors such as poor environmental conditions (e.g., worse air pollution) [43,44]. Increasing economic insecurity and precarity may directly harm health through increasing biological and psychosocial stress [40,45,46].

The diverging fortunes between the “haves” and “have-nots” may change or constrain the way individuals think and plan for the future, leading to underinvestment in behaviors that may improve health and economic outcomes. For example, worsening economic outcomes can diminish one’s expectations for a better future, which can undermine individuals’ motivations to engage in health-promoting behaviors [47–50]. In addition, economic insecurity may reduce the mental bandwidth needed to make productive, future-oriented economic decisions, reducing individuals’ ability to exit their current economic circumstances and thereby contributing to their worsening health [51,52].

### Empirical evidence linking worsening economic outcomes to population health

Descriptive evidence on trends in life expectancy and mortality by socioeconomic status implicate changing economic conditions as a key driver of worsening population health in the US. Most starkly, worsening health outcomes in the past 40 years have been concentrated among working-age adults in the bottom of the income distribution and among those with relatively low levels of formal education (i.e., high school or less). These are the very groups that have increasingly fallen behind in economic outcomes since the 1980s [1,3]. For example, a recent study using data from 1.4 billion individual-level tax records linked to administrative data on mortality found widening income-based mortality gaps from 1999 to 2014 [4]. Life expectancy increased by approximately 2–3 years over the 15-year study period for men and women with pre-tax incomes in the top 5% nationally. By contrast, life expectancies for men and women with incomes in the bottom 5% remained virtually unchanged. The growth in the mortality gap by level of education has been even more striking, with several studies demonstrating increases in mortality (or decreases in life expectancy) for adults in the bottom of the US education distribution [3, 53–56]. Growing mortality gaps by education remain [57], even after accounting for bias from growing selection into lower levels of education over time [58].

**Fig 2:**
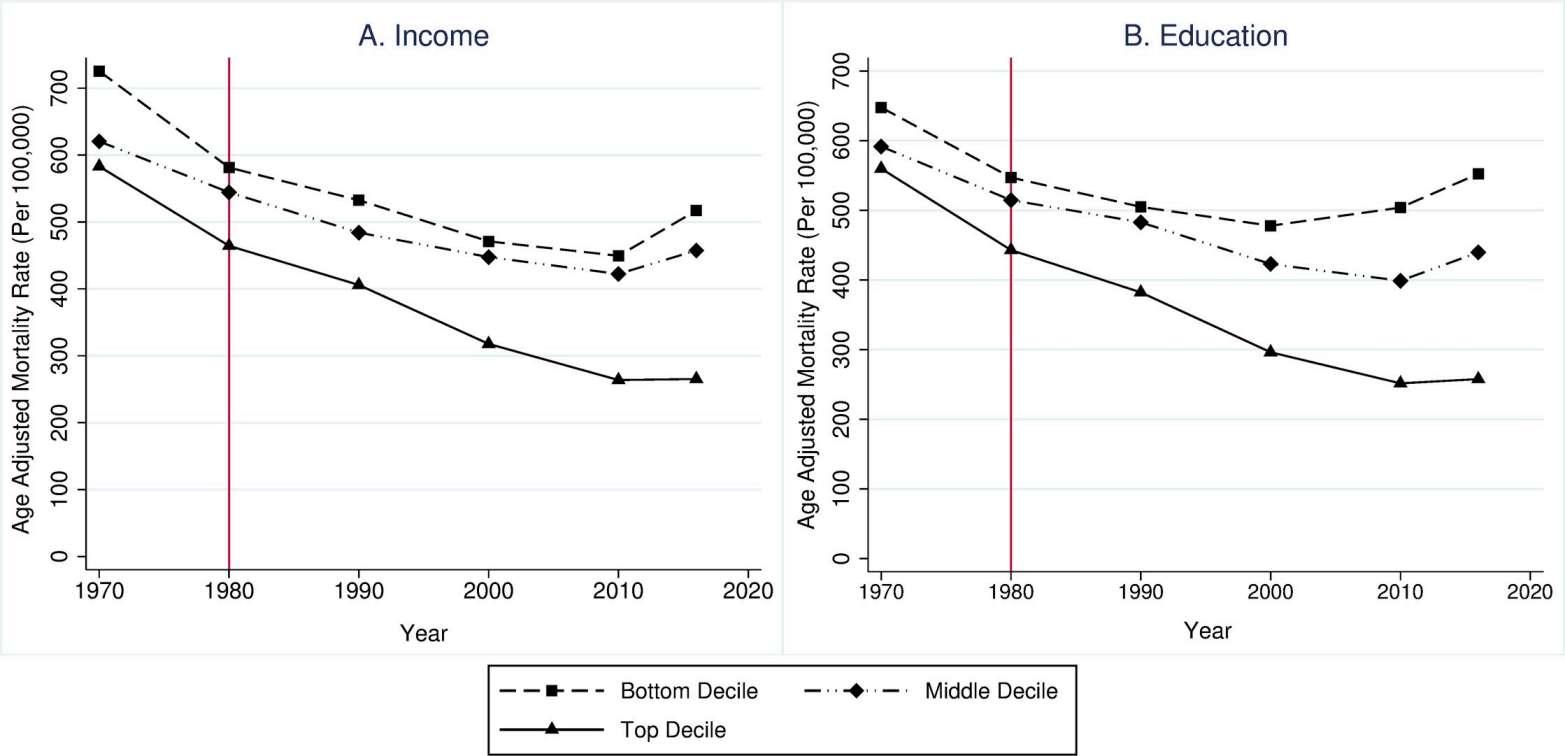
Age-adjusted mortality rates for working-age adults (ages 25–64 years) by county of residence per capital income and educational deciles, 1970–2016. County-level age-adjusted, all-cause mortality rates for adults ages 25–64 years were obtained from the US Centers for Disease Control WONDER database. Data on county-level per-capita incomes and share of individuals with some college education or greater were obtained from US Decennial Census and ACS data made available through Social Explorer. We assigned counties into deciles of the per-capita income and education variations by each census-ACS year (so a given county could be grouped into different deciles in different years). This operationalization is particularly important for level of education, as focusing on fixed educated groups (e.g., high school or less) may result in selection bias [58]. Population-weighted average mortality rates were then calculated for each socioeconomic decile-year. We then plotted these average mortality rates for the bottom (1st), middle (5th), and top (10th) decile of each socioeconomic variable. After the 1980s, the beginning of which is marked by the vertical red lines, income and education disparities began widening. ACS, American Community Survey; WONDER, Wide-Ranging Online Data for Epidemiologic Research.

Differential experiences across geographic areas are also consistent with economic factors playing a critical role in driving population health outcomes. This phenomenon can be revealed by even cursory inspection: for example, age-adjusted mortality rates in counties in different income and education deciles tracked closely with each other until the 1980s, after which the richest and more highly educated counties experienced much larger declines in death rates (Fig 2). Excess all-cause and drug overdose mortality relative to historical trends has increased in states where employment opportunities for less-educated workers have fallen the most in recent decades [1]. Areas with lower economic opportunity (operationalized as county-level differences in rates of upward mobility for individuals born into poorer families) tend to have higher mortality and morbidity [49,59,60]. Similarly, an extensive literature has demonstrated associations between rising income inequality and worsening health [61,62] (although other studies have challenged these findings [63]). Socioeconomic gaps in longevity even vary within geographic areas, with gaps growing markedly in some areas (e.g., the South and industrial Midwest) relative to others [4,54]. Metropolitan areas with higher proportions of college-educated individuals, lower unemployment, richer tax bases, and higher social mobility also tend to have narrower income-based gaps in health outcomes [4,64].

Studies examining the health consequences of specific economic insecurities among low-income and less-educated adults provide still more supporting evidence. Many of these studies have the important advantage of leveraging “natural experiments,” which are research designs that use sudden unanticipated events or shifts in policy to account for unmeasured factors that may bias estimates of the associations between economic outcomes and health [65], allowing researchers to gain more purchase in assessing causal relationships [66] and disentangling age versus period versus cohort-based explanations [67,68]. Recent studies have used policy-driven increases in local exposure to foreign trade to investigate the effects of fading economic opportunities in sectors such as manufacturing, finding large impacts on drug overdose mortality [69,70]. Other studies have demonstrated worsening mental health and increases in overall and drug overdose mortality after manufacturing plant closures [71–73]. Falling participation in labor unions has been linked to rising mortality rates from suicide and drug overdose [74]. Structural racism—as manifest by racial gaps in economic opportunity, racial gaps in wealth, racial segregation, and risk of incarceration—have also been negatively associated with a range of health outcomes, particularly for Black Americans [60,75–77].

A growing literature has also investigated the mechanisms that plausibly may link worsening economic outcomes to health. Demonstrating that key hypothesized mediators influence health provides even greater confidence that economic changes in the past 40 years have adversely affected health. Several studies have examined the health consequences of increasing precarity: food, housing, and economic insecurity, for example, have been robustly linked to a range of health outcomes, including mortality [78–80]. A number of studies have demonstrated negative effects of large economic shocks (e.g., financial hardship during the Great Recession or sudden, unexpected loss of wealth) on physiological stress as measured by cortisol, blood pressure, and blood glucose [46,81], and on premature death [82].

Our review of the evidence thus far has focused primarily on short- and medium-run impacts of economic shifts on the working-age individuals who are directly affected. However, worsening economic conditions may also negatively affect the health of those exposed to these changes early in life, prior to entering the labor market. Young adults entering the US job market during economic downturns are more likely to earn lower lifetime wages, engage in health risk behaviors such as smoking and excessive alcohol use, and, by their late 30s, have higher mortality risk, compared with their counterparts entering the labor market during better times [83,84]. Similarly, income inequality and fading economic opportunities have been linked to risky health behaviors and lower educational investments among adolescents, both of which influence health later in adulthood [85,86]. A large, robust literature has found strong links between exposure to economic insecurity in early childhood and a range of adult health outcomes [87–89].

## Social and economic policies to address population health challenges

### Evidence to date

Existing research highlights a number of social and economic policies that may jointly improve economic and health outcomes. Intervening in infancy and childhood appears to be most effective in achieving these objectives, and consequently, such interventions tend to have the highest social returns on investment [90]. The collective impacts of early investments made to date may explain why socioeconomic gaps in mortality rates for children, adolescents, and young adults have actually narrowed over the same period during which they have widened for working-age adults [91].

Evidence from randomized and natural experiments demonstrates that investments made before the age of 5 can lead to improved economic and health outcomes in adulthood [92–94]. Policies that provide early-life access to high-quality preschool and healthcare (e.g., through expansions of the Medicaid program) and reduce early exposure to pollution have also been shown to improve educational attainment, income, and health in adulthood [95–100]. Investments made later in childhood, adolescence, and young adulthood can also have large impacts on economic and health outcomes. Policies that enable children to grow up in better neighborhoods may be particularly transformative: children under the age of 13 whose families were randomly assigned to receive vouchers to move to low-poverty neighborhoods as part of the US Housing and Urban Development’s Moving to Opportunity (MTO) experiment in the late 1990s were more likely to attend and complete college and achieve incomes that were over 30% higher, compared with children whose families did not receive vouchers [101]. Researchers strongly agree on the value of increasing access to high-quality education, with reinforcing investments made throughout the K–12 years appearing to have the greatest impact in reducing economic inequality [102]. In an economy where a 4-year college education increasingly demarcates economic success from economic failure, it will be important to ensure access to high-quality colleges and universities [93,94]. The positive long-run health consequences from investing in education—particularly, college education—have been noted in a number of studies [53,84,92,103]. It will also be important to bolster opportunities for those for whom a 4-year college degree may not be a good fit, for example, through increasing access to high-return vocational education [104].

There is comparatively less evidence to inform the deployment of policy interventions aimed at working-age adults to address worsening economic outcomes and population health. Whatever evidence exists, however, suggests that the right approach will likely involve bundling multiple strategies. There is emerging evidence that minimum wage increases and the Earned Income Tax Credit result in fewer deaths from suicide among individuals with lower levels of education [105–107]. Policies and programs that assist workers displaced by automation and trade to acquire new skills may also play an important role. More generous unemployment insurance benefits have been found to mitigate the impact of economic downturns on suicide mortality [108] and overall self-reported health [109]. A recent study of the US Trade Adjustment Assistance Program, which aims to retrain workers who lose their jobs in industries affected by foreign trade, demonstrates large positive effects on employment and income [110]. Studies examining the consequences of similar policies in Europe also find important positive health impacts [111]. Recent evidence also suggests that adults moving to better neighborhoods as part of the MTO experiment achieved better health outcomes, despite little change in their economic outcomes [112]. At present, programs that foster geographic mobility and financial support to obtain new skills after job displacement remain relatively small in scope. This early evidence suggests value in scale-up and further testing.

For historically marginalized population groups such as Black Americans, improving health outcomes may additionally require supplementing universal economic and social policies with specific interventions to reduce long-standing, historically patterned barriers to social mobility. Longevity gaps between Black and White Americans persist and closely track with similar gaps in economic opportunity [60]. The positive impacts of Civil Rights–era policies on short- and long-run labor market outcomes and health [113,114] should motivate investment in a broad set of next-generation policies that include reforming law enforcement, the judicial system, and corrections; enhancing access to and affordability of high-quality education; implementing interventions aimed at narrowing the racial wealth gap; and reducing discrimination in labor markets [115–118].

It will also be important to invest in social policies that break the link between worsening economic outcomes and worsening health. A growing body of research demonstrates that expansions of state Medicaid programs have both reduced debt-driven economy insecurity among low-income adults and improved health outcomes [119–121]. Bolstering other safety net programs—e.g., Temporary Assistance for Needy Families, the Supplemental Nutritional Assistance Program—and expanding access to federal housing programs can help reduce food and housing insecurity during hard times [122]. These efforts should explicitly address administrative features that may introduce inconveniences and stigma that may discourage participation even in generous social programs [123]. Additionally, there is growing evidence that eviction and foreclosure prevention policies may have an important role to play in severing the link between adverse economic shocks and semipermanent declines in health and well-being [80,124,125].

### Future policy frontiers

The size and scope of population health declines among low-income and less-educated working-age adults also call for identifying, developing, and testing new intervention approaches. The threat of continued job displacement from automation has sparked interest in Universal Basic Income (UBI) cash-transfer programs. Early evidence from universal income programs (including ongoing programs in Alaska and the Eastern Cherokee nation) suggest positive impacts on health and educational attainment (without any evidence of the program inducing exit from the labor force) [126]. These findings reinforce earlier work showing how income supplements can improve health and developmental outcomes among children [127,128]. Large experimental evaluations of newly instituted UBI programs across the world are currently underway. Researchers and policymakers have also proposed a number of other novel transfer programs, such as automatic stabilizers to help decouple business cycle fluctuations from one’s economic circumstances [129] and reparation policies to close structurally mediated racial gaps in wealth [130]. It will also be important to consider new labor market interventions. For example, ongoing threats to economic opportunities due to automation could motivate policies that direct investment toward artificial intelligence that complements, rather than replaces, workers [131]. Job guarantee programs, which seek to provide voluntary public-sector work opportunities to those in need, have also been suggested as a potential policy option [132], particularly given positive early evidence from India [133].

Innovations in public health and healthcare systems—specifically those that help buffer population health against threats from adverse economic shocks—should also be explored. For example, there is growing interest in addressing social determinants of health among state Medicaid programs and hospital systems. Specific pilot programs (such as providing housing, community health worker programs, or interventions to increase access to other social programs) have been shown to be effective in this regard [134–137], though the evidence base as a whole remains sparse. In addition, heightened screening and surveillance in areas facing economic downturns, empowering healthcare practitioners to identify social drivers of health and refer patients to local resources, and fostering partnerships between community-based organizations and health systems may represent a comprehensive and cohesive action plan to mitigate the negative consequences of worsening economic outcomes [138–141]. Implementation and scale-up of these interventions may require redesigning financial incentives in healthcare in ways that direct investment toward addressing socioeconomic determinants of health, e.g., alternative payment models or financial partnerships between healthcare and social sector organizations to address key social issues affecting health and economic outcomes within communities [141,142]. It will also be important to ensure programs implemented by healthcare organizations do not crowd out or replace similar, but higher-return, efforts by other organizations (e.g., public health or housing departments).

## Investing in new data and enhancing data access to inform policy

A detailed understanding of how the changing economy has affected population health has been made possible by newly available large administrative databases and innovations in statistical techniques. New insights on the changing relationship between income and health have come from combining data from tax databases with mortality data from Social Security death records [4]. Evidence on the impacts of the US Trade Adjustment Assistance program on the incomes of workers comes from unprecedented linkages between US Census data and program administrative data [110]. Landmark contributions on the impacts of environmental policies on health and economic outcomes result from combining individual- and firm-level employment and wage data collected by states with detailed local-area data on air pollution [98]. Researchers studying the impacts of specific social and economic policies on a range of health outcomes have creatively combined hand-collected information on the timing of policy adoption with data on health outcomes from publicly available data from long-standing surveys, such as the Behavioral Risk Factor Surveillance Study and National Health Interview Survey, or vital statistics data [72,86,106,143].

Although many of these novel datasets have been gathered or built by individual research groups, there is an increasing trend toward data sharing, further catalyzing research on the economic determinants of health. For example, researchers releasing deidentified, aggregate versions of their tax-record data have enabled other scholars to identify new insights on the relationship between economic opportunity and health [50,64,144,145]. The growth of federally and privately funded “data aggregators” (e.g., the Integrated Public Use Microdata Series [IPUMS] or Inter-university Consortium for Political and Social Research [ICPSR]) has also been important for researchers studying the economy and health. Data aggregators serve a variety of functions, including enabling easy access to cleaned and harmonized census, health, and economic survey data; collating and disseminating data used in other academic papers; and collecting vetted information on policy implementation across a variety of domains. State and federal government databases are becoming increasingly more complete and easier to access through public-facing web portals, with prime examples being the US Centers for Disease Control Wide-Ranging Online Data for Epidemiologic Research (WONDER) database for vital statistics and the US Bureau of Labor Statistics databases for unemployment and income data.

Despite these encouraging trends, researchers and policymakers still face significant challenges obtaining the data needed to best inform policymaking. Large, annual sample surveys primarily collect detailed information on health outcomes or economic outcomes, but not both, making it difficult to study the nuances of how economic trends shape various dimensions of health in different populations. Linking large-scale, individual-level federal economic datasets to health datasets such as death records or medical claims remains a costly, time-consuming process for most researchers. As a result, researchers studying the health consequences of adverse economic trends have had to rely on aggregate data, which are prone to their own biases (e.g., researchers cannot reliably account for nonrandom migration) and make it difficult to study heterogeneity across population groups. Moreover, most of these data are released from 1–2 years after collection, making it difficult to provide real-time information on key questions of interest.

The increasing availability of proprietary private data sources and high-frequency, crowd-sourced data has helped address some, but unfortunately not all, of these constraints. Consequently, new policies to increase access to existing large-scale administrative and survey data are needed to maximize our understanding of what policies are most effective in addressing the twin challenges of worsening economic outcomes and population health in the US. Steps to reduce administrative and financial barriers to accessing and linking state and federal data resources will be critical. In this respect, state and federal agencies could follow the lead of the National Institutes of Health (NIH), who recently made data linkages with the death registration system free for NIH-supported researchers, streamlined the process to apply for these data and increased the frequency at which these data were updated. Insights from the development and management of wide-ranging linked registries, such as those in Sweden or Denmark, could also provide a useful model. Agencies can also consider making some currently restricted-access data (e.g., vital statistics for counties with small numbers of births or deaths) public by adopting newly developed techniques to add noise to these measures in a manner that reduces loss of privacy while maintaining statistical fidelity [146].

## Evidence-based policy and politics

The full potential of data-driven policymaking to improve economic and health outcomes cannot be realized without buy-in from policymakers. There needs to be consensus and political will among policymakers around investing in data and acting on evidence. Translating evidence into policy will require a detailed understanding of the political considerations, policymaker values, and decision-making processes that influence policy adoption [147,148]. Evidence may be valued differently by different actors and in different circumstances [149–151]. Wherever it is possible to do so, collaboration between researchers and policymakers to incorporate data collection efforts and rigorous evaluation designs as part of the policy implementation process [151,152] will be important to help build consensus around the value of data and evidence-based policymaking.

In addition, it will be important to elucidate the complex feedback loops between health, economic outcomes, and voting patterns that will ultimately dictate which policies are adopted [147]. Exposure to international trade, which has been tied to worse economic outcomes and deteriorating health among working class individuals [15,70], has led to increased support for presidential candidates whose policy agendas have often run counter to emerging evidence on how to improve economic well-being and population health [153]. These dynamics were particularly salient in the 2016 presidential election, in which areas with worsening health outcomes saw increased support for Donald Trump [154,155], a candidate who campaigned, for example, on deconstructing the Affordable Care Act and reducing environmental regulation. On the other hand, expansion of the Medicaid program, which has shown to improve both health and economic outcomes among low-income US residents [99,119,120], was associated with increased support for the program [156] and greater voter turnout [157]. Incorporating the study of such “political feedback loops” into analyses of the effects of social and economic policies will thus be important to fully elucidate their long-run economic and health consequences.

## Conclusion

Population health in the US stands at a critical juncture. Growing evidence suggests that fading economic opportunities and rising economic insecurity have played an important role in the deteriorating health outcomes and rising mortality rates experienced by working-age individuals. These trends may be further exacerbated by the SARS-CoV-2 pandemic, whose adverse economic consequences—and wide-ranging direct and indirect negative effects on a variety of health outcomes—are expected to most heavily affect low-income individuals. Despite these troubling trends, there are reasons for optimism. New data have enabled researchers and policymakers to better delineate emerging population health challenges and identify policies that can reduce growing health and economic inequality. Further investments in new data and ensuring better access to, and linkages between, existing databases will be necessary to fully realize the potential of evidence-based social and economic policy. However, data alone will not be sufficient. Policymaker consensus around the value of data—and the political will to act on it—will be critical for translating evidence into improvements in population health.

## Abbreviations

ACS: American Community Survey
ICPSR: Inter-university Consortium for Political and Social Research
IPUMS: Integrated Public Use Microdata Series
MTO: Moving to Opportunity
NIH: National Institutes of Health
UBI: Universal Basic Income
WONDER: Wide-Ranging Online Data for Epidemiologic Research
